# Supplementary material for: Loss of autophagy impairs physiological steatosis by accumulation of NCoR1
Source: Life Sci Alliance. 2019 Dec 26;3(1):e201900513. doi: 10.26508/lsa.201900513 (PMC6932742; doi:10.26508/lsa.201900513)
Supplement: Supplementary file 3 [file LSA-2019-00513_TableS1.doc]

Supplementary Table 1. Primer sequences used for RT qPCR.

| Gene | Left | Right |
| --- | --- | --- |
| *MmGus* | Ctctggtggccttacctga | ctcagttgttgtcaccttcacc |
| *MmLxrα* | Gagtgtcgacttcgcaaatg | cggatctgttcttctgacagc |
| *MmAcly* | Gtggccccaactatcaaagag | atgggatcccagtggtc |
| *MmAcaca* | agcaacatcacatcagtcctgt | cagtgtagctgcatgactatctagg |
| *MmFasn* | gctgctgttggaagtcagc | agtgttcgttcctcggagtg |
| *MmScd1* | ttccctcctgcaagctctac | cagagcgctggtcatgtagt |
| *MmFatp2* | gcgtgcctcaactacaacatt | cctcctccacagcttcttgt |
| *MmFatp5* | agggtttttgcattcctgtg | ttggttctttcgaaccttgg |
| *MmDgat1* | gccccatgcgtgattatt | tctgtcagggcacccact |
| *MmDgat2* | gctggtgccctactccaag | ccagcttggggacagtga |
| *MmGk* | agcagttctggggccatt | agaagttcagctgttttgaattg |
| *HsGAPDH* | acgggaagcttgtcatcaat | catcgccccacttgatttt |
| *HsACLY* | aaccccaaaggaggatct | ttgacaccccctagatcacag |
| *HsACACA* | gatgtggatgatgggctaca | tgaggccttgatcattactgg |
| *HsFASN* | gaggaggggtgcctgtct | tgggtcaccttggtctgc |
| *HsSCD1* | cctagaagctgagaaactggtga | acatcatcagcaagccaggt |
| *HsLXRα* | gttataaccgggaagacttgc | aaactcggcatcattgagttg |
| *HsFATP2* | ccagagtcatggaggtctgaa | gcaaggcaagagtagcacct |
| *HsDGAT1* | cccaacaaggacggagac | agaatcctgcaggcgatg |
| *HsDGAT2* | tactccaagcccatcaccac | ggtgtggtacaggtcgatgtc |
| *HsGK* | ggttcatggcagcctca | ctaagtagttcagctgttttgaattg |
